# Layer-sensitive cognitive offloading in generative AI-assisted writing: supported performance and independent no-AI outcomes

**DOI:** 10.3389/fpsyg.2026.1906199

**Published:** 2026-08-28

**Authors:** Xinran Chen

**Affiliations:** School of Foreign Languages, Jinggangshan University, Ji'an, Jiangxi, China

**Keywords:** generative artificial intelligence, cognitive offloading, self-regulated writing, higher-order thinking, academic writing, independent performance, learner agency

## Abstract

**Introduction:**

Writing with generative artificial intelligence (GenAI) can improve a supported product without necessarily strengthening the competence that remains after support is removed. This study examined that distinction through a layer-sensitive account of cognitive offloading.

**Methods:**

In this eight-week quasi-experimental classroom study, 180 Chinese undergraduates from six intact English academic-writing classes entered the study and 168 completed the protocol. Two classes were assigned to each of three conditions: no-AI writing, bounded AI support with compulsory reflection, and open AI collaboration. Students completed a baseline task, three intervention assignments, and a supervised Week 8 independent no-AI near-transfer task.

**Results:**

Open AI collaboration had the highest observed supported-writing mean (*M* = 4.02), although the class-clustered open–bounded contrast was imprecise (adjusted difference = 0.21, 95% CI [0.06, 0.36], wild-cluster *p* = 0.064). At Week 8, the bounded-support condition showed higher adjusted outcomes than open collaboration in writing quality (difference = 0.27), higher-order thinking (0.35), argument depth (0.42), and independent revision quality (0.39); exact wild-cluster *p* values ranged from 0.050 to 0.063. Within the 112 AI-exposed students, open collaboration involved more prompts, greater text incorporation, less self-generated text, and deeper idea and reasoning offloading. An adjusted associative decomposition showed that open collaboration predicted higher aggregate offloading (*a* = 0.77, *SE* = 0.06), which was associated with lower Week 8 higher-order thinking (*b* = −0.45, *SE* = 0.08); the bootstrap indirect estimate was −0.34, 95% CI [−0.48, −0.21]. Self-regulated writing attenuated, but did not eliminate, this negative association.

**Discussion:**

Because conditions were allocated by only six intact classes and the bounded condition combined delegation restrictions with compulsory reflection, the results are interpreted as classroom-level, mechanism-consistent associations rather than definitive causal effects. The findings distinguish performance achieved with GenAI from competence demonstrated independently after support is removed.

## 1 Introduction

Academic writing is both a product and a learning process. Students must plan ideas, construct and qualify claims, evaluate evidence, anticipate alternative views, monitor coherence, and revise meaning. These activities overlap with established accounts of critical thinking, particularly interpretation, analysis, inference, evaluation, explanation, and self-regulation (Facione, 1990). Cognitive models consequently describe writing as recursive problem solving rather than transcription (Flower and Hayes, 1981; Hayes, 2012). The educational value of writing lies not only in the quality of the submitted text but also in the cognitive operations exercised while producing it (Bereiter and Scardamalia, 1987; Kellogg, 1996; Graham and Harris, 2000; Graham et al., 2005).

Generative artificial intelligence (GenAI) changes this relationship between process and product. A student can use an AI system to brainstorm, produce an outline, draft paragraphs, generate counterarguments, restructure an essay, or polish language. Reviews and conceptual analyses identify substantial opportunities for feedback, personalization, and writing support, alongside concerns about accuracy, overreliance, academic integrity, and assessment validity (Kasneci et al., 2023; Lo, 2023; Dwivedi et al., 2023). Empirical studies further suggest that GenAI feedback can support writing performance when learners remain active in interpreting and revising the output (Warschauer et al., 2023; Mahapatra, 2024; Polakova and Klimova, 2024; Wang and Ren, 2024). These affordances can reduce linguistic barriers and provide immediate feedback. They can also make the submitted product a poor indicator of what the learner can do independently. A fluent essay may reflect active evaluation and transformation of AI feedback, or it may reflect the minimally transformed adoption of externally generated ideas and reasoning.

This distinction corresponds to two established educational-psychology principles. First, effects obtained *with* a technology are not identical to effects that remain *of* the technology after support is removed (Salomon et al., 1991). Second, performance during supported practice can improve even when durable learning does not (Soderstrom and Bjork, 2015). Accordingly, educational transfer must be evaluated after support is withdrawn rather than inferred from assisted performance alone (Perkins and Salomon, 1992). GenAI therefore requires an assessment design that separates supported performance from independent post-intervention performance. In the present study, the Week 8 task is treated as a same-course, same-genre near-transfer assessment and is described throughout as an independent no-AI posttest rather than as evidence of broad or far transfer.

Cognitive offloading provides a mechanism for explaining why supported and independent performance may diverge. Cognitive offloading is the use of external tools or environmental structures to reduce internal cognitive demand (Risko and Gilbert, 2016). Offloading is not inherently harmful. Notes, dictionaries, outlines, and feedback can reduce peripheral demands and free resources for more important reasoning, consistent with the principle that reducing extraneous cognitive load can facilitate learning (Sweller, 1988). Risk arises when the tool performs the operations through which competence is developed. Recent research has therefore begun to examine cognitive offloading as a mechanism linking AI use to critical-thinking and learning outcomes (Gerlich, 2025; Jose et al., 2025; Iqbal et al., 2025). GenAI is distinctive because it can offload not only wording and memory but also content selection, argument organization, evidence interpretation, and evaluative judgment.

The present study distinguishes four analytic layers. Surface offloading concerns grammar, vocabulary, and local expression. Structural offloading concerns outlines, sequencing, and global organization. Idea offloading concerns claims, examples, perspectives, and content directions. Reasoning offloading concerns warrants, counterarguments, evidence interpretation, and argumentative logic. These layers were treated as related but non-equivalent dimensions. The primary layer scores were derived from coded interaction and revision evidence; brief self-report indicators were used only for triangulation and were not treated as independent latent traits.

The study also distinguishes scaffolding from unrestricted delegation. Classical scaffolding is contingent, supports learner participation, and transfers responsibility to the learner as competence develops (Wood et al., 1976; van de Pol et al., 2010). The bounded-support condition in this study imposed fixed boundaries rather than adaptive fading. It therefore represents bounded instructional support, not a complete implementation of contingent scaffolding. In addition, students in that condition justified how AI suggestions were accepted, changed, or rejected. This reflective requirement is a metacognitive component bundled with the delegation boundary. The study cannot isolate its effect from the effect of the boundary itself, and the interpretation explicitly preserves that limitation.

Self-regulated writing may determine whether AI remains supportive or becomes substitutive. Self-regulated writers set goals, monitor coherence, evaluate feedback, manage effort, and retain authority over revision (Zimmerman and Risemberg, 1997; Pintrich, 2000; Panadero, 2017). In GenAI-assisted writing, these processes include deciding why AI is being consulted, checking whether an output matches the writer's intention, verifying claims, transforming suggestions, and reflecting on what was learned. These demands make GenAI use inherently metacognitive because users must monitor outputs, calibrate reliance, and decide when to accept, revise, or reject suggestions (Tankelevitch et al., 2024). Self-regulated writing was therefore examined as a moderator of the association between offloading and independent performance.

The central research questions were:

**RQ1:** How do no-AI writing, bounded AI support, and open AI collaboration differ in supported writing performance and independent no-AI posttest performance?
**RQ2:** Among students with access to AI, how do surface, structural, idea, and reasoning offloading relate to independent higher-order-thinking performance?
**RQ3:** Does self-regulated writing attenuate the association between deeper cognitive offloading and independent higher-order-thinking performance?
**RQ4:** What prompt, adoption, revision, and textual patterns distinguish bounded support from more substitutive AI use?

## 2 Literature review

### 2.1 Writing, supported performance, and learning

Writing requires coordination among planning, translation, review, working memory, knowledge retrieval, and rhetorical decision making (Flower and Hayes, 1981; Kellogg, 1996; Hayes, 2012). A writing intervention can therefore improve a product through at least two pathways. It can strengthen the learner's internal strategies, or it can provide external support that raises performance while the support is present. These pathways may coexist, but they are not interchangeable. The performance–learning distinction is especially important when a tool can produce complete language and reasoning structures (Soderstrom and Bjork, 2015).

Salomon, Perkins, and Globerson's distinction between effects with and effects of technology is directly relevant (Salomon et al., 1991). GenAI-supported writing reveals what students and systems can produce together. An independent no-AI task reveals what students can produce after the system is removed. The latter does not establish far transfer, but it provides a closer indicator of internalized course-relevant performance than an AI-supported product alone.

### 2.2 Layer-sensitive cognitive offloading

Cognitive offloading can improve immediate performance by externalizing memory, computation, or planning (Risko and Gilbert, 2016). It can also reduce later retention or internal processing when learners cease to encode or practice the offloaded operation (Sparrow et al., 2011; Grinschgl et al., 2021). Whether offloading supports learning depends on the operation being delegated and on the learner's continuing cognitive participation.

In academic writing, surface support may reduce L2 language burden without displacing argument construction. Structural support is more ambiguous because an outline can function either as a prompt for evaluation or as an adopted architecture. Idea and reasoning offloading reach the substance of knowledge transformation. A student who adopts claims, warrants, evidence interpretations, or counterarguments has fewer opportunities to practice the operations that an independent writing task later requires. For this reason, frequency alone is insufficient: one reasoning-generation request may carry greater developmental significance than several requests for local language clarification.

### 2.3 Scaffolding, reflection, and responsibility

Scaffolding is effective when support is contingent on need, maintains learner participation, and is gradually withdrawn (Wood et al., 1976; van de Pol et al., 2010). GenAI can support these functions when it asks diagnostic questions, identifies weaknesses, or offers alternatives that students must evaluate. It becomes more substitutive when it supplies ready-to-use arguments or prose with little transformation.

The present bounded-support condition combined two components: restrictions on the depth of delegation and compulsory explanation of how AI suggestions were adapted, rejected, or transformed. The explanation requirement plausibly increases metacognitive monitoring. Consequently, any advantage of the bounded-support condition must be interpreted as the effect of a bundled instructional design rather than as a pure effect of delegation boundaries.

### 2.4 Self-regulated writing in L2 GenAI use

Self-regulated writing includes planning, monitoring, feedback evaluation, strategic revision, and reflection (Zimmerman and Risemberg, 1997; Graham and Perin, 2007). These processes are particularly important for L2 writers because linguistic fluency can mask weak reasoning, while GenAI can improve language rapidly. The participants in this study were Chinese L1 undergraduates writing in English. General writing quality and higher-order thinking were therefore scored separately, and baseline English writing performance was included in adjusted analyses.

AI-specific verification items were not included in the cross-condition self-regulated-writing score because they were inapplicable to the no-AI group. Cross-group analyses used only a common core of planning, monitoring, feedback evaluation, and revision-control items. AI-output verification was analyzed descriptively within the two AI conditions.

## 3 Method

### 3.1 Participants, class allocation, and design sensitivity

Participants were 180 undergraduates enrolled in six intact English academic-writing classes at Jinggangshan University. Each class initially included 30 consenting students. Twelve students were excluded because of absence from the Week 8 assessment or incomplete records required for the prespecified analyses. Attrition was balanced across conditions and classes: two students were excluded from each class, leaving 168 participants, 56 per condition and 28 per class (Table 1).

**Table 1:** Class-level allocation, instructor balance, and participant flow.

Condition	Class	Instructor	Entered	Excluded	Analytic sample
No-AI writing	N1	A	30	2	28
No-AI writing	N2	B	30	2	28
Bounded AI support	S1	A	30	2	28
Bounded AI support	S2	B	30	2	28
Open AI collaboration	O1	A	30	2	28
Open AI collaboration	O2	B	30	2	28
Total	6 classes	2 instructors	180	12	168

Two instructors each taught one class in every condition. Classes used the same syllabus, task windows, prompts, rubrics, platform instructions, and assessment timetable. Nevertheless, condition was assigned to intact classes rather than individual students. Condition therefore remained inseparable from class-level influences such as timetable and cohort composition. The study is consequently described as quasi-experimental, and the primary uncertainty estimates respect class clustering.

An individual-level planning calculation for a three-group comparison with *f* = 0.25, α = 0.05, and 0.80 power indicated a minimum of 159 students; the recruitment target was 180. This calculation did not solve the design limitation created by six assignment units. In the AI-exposed subsample (*n* = 112), a covariate-adjusted regression had approximately 0.80 power to detect a partial correlation of about 0.27. Indirect and interaction analyses were therefore designated exploratory. No analysis treats the student count as compensation for the small number of classes.

All participants were native speakers of Chinese and completed every essay in English. Baseline English writing performance was used as the direct L2 proficiency covariate. Written informed consent was obtained before data collection.

### 3.2 Instructional sequence and conditions

The eight-week sequence comprised a supervised baseline no-AI task, three intervention writing cycles, and a supervised Week 8 independent no-AI assessment. Every writing task used a staged process: an initial draft was saved before revision, followed by a revised final submission. The three conditions were:

- No-AI writing: students planned, drafted, and revised without GenAI or automated rewriting tools.
- Bounded AI support: students could request brainstorming questions, alternative outline options, reader-response feedback, identification of weaknesses, local coherence advice, and language clarification. Requests for complete paragraphs, final thesis statements, complete argumentative structures, ready-to-use evidence interpretations, full counterarguments, or full-text rewriting were prohibited. For each AI-influenced change, students recorded whether the suggestion was accepted, modified, rejected, or used only to stimulate further thinking, and explained the decision.
- Open AI collaboration: students could use the same platform for planning, drafting, argument development, counterargument generation, global revision, and language polishing, provided that all interactions and adoption decisions were logged.

The bounded condition was a bundled instructional treatment: it combined limits on delegation depth with compulsory metacognitive explanation. The design cannot identify the independent effect of either component. Within the bounded condition, coded reflective engagement was analyzed as a mechanism-consistent predictor rather than as a randomized mediator.

### 3.3 GenAI platform and data-collection window

Data collection ran from 2 March to 24 April 2026. AI-supported tasks were completed from 9 March to 17 April 2026 using ChatGPT with GPT-5.3 through the official browser interface. Students used ordinary text-chat mode, opened a new conversation for each task, and did not use custom agents, model switching, file uploads, third-party writing extensions, or external paraphrasing tools. Browser-exposed sampling controls were unavailable. Prompt–response histories were exported immediately after each task.

### 3.4 Baseline higher-order-thinking pretest

The higher-order-thinking pretest was the Baseline No-AI Argumentation Task. Students wrote a 450–550-word English argumentative essay under supervised conditions for 60 min without internet access, GenAI, previous writing, notes, or course materials. The prompt required a defensible position, at least two reasons, integration of examples or evidence, and consideration of a counterargument. Students saved an initial draft after 35 min and used the remaining 25 min for revision. The same four-dimension higher-order-thinking rubric used for subsequent tasks was applied to this pretest.

### 3.5 Week 8 independent no-AI assessment

Students were informed at the beginning of the study that the sequence would end with an independent assessment, but neither the topic nor source material was disclosed. The Week 8 task was administered in a supervised computer room for 60 min. Students used a blank word-processing file and could not access GenAI, the internet, earlier essays, AI histories, course notes, dictionaries, or course materials. An initial draft was saved after 35 min; students then revised for 25 min, allowing independent revision quality to be evaluated from the paired versions.

Parallel difficulty was established before data collection by matching the intervention and Week 8 prompts on argumentative genre, 450–550-word range, required number of claims, evidence-integration demand, counterargument demand, and rubric coverage. Two English-writing instructors independently reviewed the prompts and resolved discrepancies before administration. The Week 8 topic differed from all intervention topics. Because the assessment remained within the same course and genre and immediately followed the intervention, it is interpreted as near transfer and independent post-intervention performance, not broad or far transfer.

### 3.6 Data construction

Table 2 makes the corpus arithmetic explicit. The 840 final writing products comprise five tasks for each of 168 students. Each task also produced a saved draft–final pair. The writing-quality rubric generated 4,200 dimension scores (840 × 5), and the higher-order-thinking rubric generated 3,360 dimension scores (840 × 4), before averaging the two raters. The two AI conditions contributed 336 task-level AI-use records (112 × 3). The 1,273 bounded-condition and 2,339 open-condition prompt turns were verified from 336 integer-valued student–task records and correspond to 7.58 and 13.92 prompts per student per task, respectively. A range audit confirmed that all public 1–5 measures fell within their declared bounds. Two post-de-identification boundary corrections are documented in the supplementary correction log and were verified by the research team against the original scoring sheets and raw export records. Measurement quality and construct-separation evidence for the principal measures are summarized in Table 3.

**Table 2:** Data construction and corpus profile.

Data source	Participants	Records per participant	Expected total	Valid total	Analytic role
Final writing products	168	5	840	840	Baseline, supported, and Week 8 outcomes
Draft–final pairs	168	5	840	840	Revision and process scoring
Writing-quality dimensions	168	5 × 5	4,200	4,200	Five dimensions per task
Higher-order-thinking dimensions	168	5 × 4	3,360	3,360	Four dimensions per task
Intervention questionnaires	168	3	504	504	Process measures
AI-supported task records	112	3	336	336	Prompt and adoption coding
Bounded-condition rationales	56	3	168	168	Reflective engagement
Open-condition AI-use summaries	56	3	168	168	Adoption and ownership coding
Prompt turns: bounded AI	56	3 tasks	—	1,273	Mean = 7.58 per task
Prompt turns: open AI	56	3 tasks	—	2,339	Mean = 13.92 per task
Total prompt turns	112	3 tasks	—	3,612	Prompt-function corpus

**Table 3:** Measurement quality and construct-separation evidence.

Measure	Reliability/agreement	Structural evidence	Cross-source/construct evidence	Analytic use
Writing quality	ICC = 0.83–0.85	Five rubric dimensions	Correlation with HOT: *r* = 0.63	Product outcome
Higher-order thinking	ICC = 0.83–0.88	Four rubric dimensions	Argument quality–depth: *r* = 0.66	Reasoning outcome
Layered offloading	κ = 0.79–0.86; α = 0.81–0.85	Four-factor CFI = 0.959, RMSEA = 0.054	Coded–self-report *r* = 0.66–0.77	Process indicators
Common SRW score	α = 0.90–0.92	Scalar CFI = 0.976, RMSEA = 0.034	Same eight items in all groups	Moderator/covariate

### 3.7 Measures and measurement quality

#### 3.7.1 Writing quality and rater blinding

Writing quality was scored from 1 to 5 on content development, argument quality, organization, language use, and coherence. Two trained raters who did not teach any participating class independently evaluated all texts. Files were de-identified, stripped of condition labels and process records, and presented in randomized order. The Week 8 essays were scored in a separate randomized batch. Raters were blind to assigned condition, although two intervention topics concerned AI or online learning and could have permitted imperfect inference from content. Disagreements exceeding one scale point were adjudicated without revealing condition.

Two-way random-effects absolute-agreement ICCs were 0.83 at baseline, 0.85, 0.84, and 0.83 across the intervention assignments, and 0.85 for Week 8 writing.

#### 3.7.2 Higher-order thinking and construct separation

Higher-order thinking was scored from 1 to 5 on argument depth, evidence integration, evaluative judgment, and reflective revision. The same blinded procedure was used. ICCs were 0.83 at baseline, 0.86, 0.88, and 0.87 across intervention assignments, and 0.85 at Week 8.

At Week 8, writing quality and higher-order thinking correlated at *r* = 0.63. Argument quality and argument depth correlated at *r* = 0.66, and the two revision-related scores correlated at *r* = 0.58. These moderate associations indicate overlap expected of related writing constructs but not score identity. Higher-order-thinking models adjusted for baseline writing proficiency, and language use was never treated as direct evidence of reasoning.

#### 3.7.3 Layer-specific cognitive offloading

The primary offloading indicators were coded from prompts, AI outputs, adoption decisions, and draft–revision records. Surface, structural, idea, and reasoning offloading were scored from 1 (the operation remained student-performed) to 5 (the operation was predominantly externally performed and minimally transformed). Two coders independently coded 84 of the 336 AI-supported task records (25%) before consensus resolution. Weighted kappas were 0.82, 0.83, 0.79, and 0.86 for surface, structural, idea, and reasoning offloading.

A parallel 16-item self-report instrument contained four items per layer and was used to examine the structure and convergence of the coding framework. Coefficients α were 0.85, 0.82, 0.81, and 0.85. A four-factor model fitted substantially better than a one-factor model, with all item wording, standardized loading output, factor correlations, reliability estimates, and analysis syntax supplied in the Supplementary material: four-factor χ^2^(98) = 129.37, *p* = 0.019, CFI = 0.959, TLI = 0.950, RMSEA = 0.054, SRMR = 0.071; one-factor χ^2^(104) = 420.38, CFI = 0.585, TLI = 0.521, RMSEA = 0.166, SRMR = 0.133. Condition-adjusted correlations among coded layers ranged from 0.49 to 0.67. Coded–self-report convergence correlations were 0.73, 0.70, 0.66, and 0.77. The coding and self-report measures are therefore reported separately: the former are the primary process indicators, and the latter provide structural and convergent evidence.

#### 3.7.4 Self-regulated writing

The cross-condition self-regulated-writing score used eight common items concerning goal setting, planning, coherence monitoring, feedback evaluation, revision control, reason-giving, and post-task reflection. Three AI-output-verification items were administered only to the AI conditions and were excluded from all three-condition comparisons.

Internal consistency was α = 0.90, 0.92, and 0.92 in the no-AI, bounded, and open conditions. Multi-group CFA supported configural, metric, and scalar comparability of the common scale. Configural fit was χ^2^(60) = 77.77, CFI = 0.976, TLI = 0.966, RMSEA = 0.042; metric fit was χ^2^(74) = 90.33, CFI = 0.978, TLI = 0.975, RMSEA = 0.037; scalar fit was χ^2^(88) = 105.26, CFI = 0.976, TLI = 0.977, RMSEA = 0.034. Relative to the metric model, scalar constraints changed CFI by −0.001 and RMSEA by −0.002; both differences were calculated from the unrounded fit indices. The same eight items were therefore used for observed-score comparisons across conditions.

### 3.8 Practice-volume and reflective-engagement indicators

Self-generated text proportion was estimated by aligning AI outputs with final submissions and classifying unmatched student-authored material after manual verification. Active drafting minutes were derived from first and final save times after removing documented breaks longer than ten minutes. These measures were treated as process indicators rather than exact keystroke records. Reflective engagement in the bounded condition was coded from 1 to 5 according to the specificity of the adaptation rationale, evaluation of fit, and evidence of an independent decision.

### 3.9 Analysis strategy

Baseline equivalence was described with student-level summaries because it concerned observed sample composition. For supported outcomes, the three repeated assignments were modeled with condition, centered assignment, and their interaction, adjusting for baseline writing. For Week 8 outcomes, adjusted linear models included baseline writing, higher-order-thinking pretest, prior AI experience, and writing self-efficacy. In both cases, inference used small-sample-corrected class-clustered covariance with *t*(5) confidence intervals and an exact Webb six-point wild-cluster reference distribution enumerating all 46,656 class-weight combinations. These procedures improve finite-sample reporting but do not recover information unavailable from only six clusters.

Prompt frequency and text incorporation were structural zeros in the no-AI condition and were therefore compared only between the two AI conditions using Welch tests and Hedges' *g*. Layer comparisons and all mechanism analyses were likewise restricted to the 112 AI-exposed students. Aggregate and layer-specific associative decompositions reported *a*, *b*, *c*, and *c*′ paths, standard errors, *p* values, mediator and outcome *R*^2^, and 5,000 bootstrap intervals for indirect estimates. The moderation model mean-centered aggregate offloading and common self-regulated writing. Reflective engagement was examined only within the bounded group. A supplementary practice-volume model entered self-generated text proportion alongside offloading. These mechanism models are exploratory and do not establish causal mediation.

## 4 Results

### 4.1 Baseline comparability

The conditions showed no large baseline imbalance (Table 4). These student-level summaries do not remove class-level confounding.

**Table 4:** Baseline characteristics across instructional conditions.

Variable	No-AI (*n* = 56)	Bounded AI (*n* = 56)	Open AI (*n* = 56)	*F* / χ^2^	*p*	Effect size
Baseline writing proficiency	3.22 ± 0.40	3.25 ± 0.38	3.20 ± 0.41	0.23	0.799	η^2^ = 0.003
Higher-order-thinking pretest	3.16 ± 0.43	3.21 ± 0.39	3.17 ± 0.42	0.23	0.796	η^2^ = 0.003
Writing self-efficacy	3.45 ± 0.54	3.50 ± 0.52	3.44 ± 0.56	0.20	.820	η^2^ = 0.002
Prior AI experience	2.92 ± 0.72	2.90 ± 0.68	3.08 ± 0.70	1.11	0.331	η^2^ = 0.013
GPA	3.30 ± 0.35	3.33 ± 0.34	3.28 ± 0.36	0.29	0.749	η^2^ = 0.003
Female, *n* (%)	35 (62.5)	34 (60.7)	36 (64.3)	0.15	0.927	*V* = 0.030

### 4.2 Supported writing performance

Observed supported-writing means increased across assignments in all conditions and were highest in open collaboration (Table 5). On the aggregated student score, open collaboration exceeded bounded support by 0.19 points (Hedges' *g* = 0.66), bounded support exceeded no-AI writing by 0.26 (*g* = 0.87), and open collaboration exceeded no-AI writing by 0.45 (*g* = 1.48). These effect sizes describe the student distributions and are not the primary class-level tests.

**Table 5:** Observed supported writing quality during the intervention.

Condition	Assignment 1	Assignment 2	Assignment 3	Aggregated mean
No-AI writing	3.45 ± 0.36	3.56 ± 0.34	3.69 ± 0.33	3.57 ± 0.32
Bounded AI support	3.70 ± 0.33	3.83 ± 0.29	3.96 ± 0.28	3.83 ± 0.28
Open AI collaboration	3.89 ± 0.34	4.02 ± 0.30	4.15 ± 0.29	4.02 ± 0.29

The adjusted class-clustered open–bounded contrast at the midpoint assignment was 0.21, *SE* = 0.06, 95% CI [0.06, 0.36], with wild-cluster *p* = 0.064. The bounded–no-AI and open–no-AI changes over assignment were both 0.01 points per task, 95% CI [−0.03, 0.05], exact wild-cluster *p* = 0.644 for both contrasts. The open–bounded difference in change was effectively zero, 95% CI [−0.03, 0.03], *p* = 1.000. An omnibus two-degree-of-freedom cluster-robust Wald test likewise found no overall condition × assignment interaction, *F*(2, 5) = 0.30, *p* = 0.754. Thus, the observed ordering was stable across tasks, but the six-class inference for the open–bounded supported-performance contrast remained imprecise.

### 4.3 AI-use intensity, incorporation, practice volume, and revision ownership

The no-AI condition was excluded from variables that were zero by design. Open collaboration involved more prompts, greater AI-text incorporation, less independent revision, less self-generated text, and shorter active drafting time (Table 6).

**Table 6:** Process indicators within the AI-exposed subsample.

Indicator	Bounded AI	Open AI	Open–bounded	Welch *t*(*df*)	95% CI	Hedges' *g*
Aggregate offloading	3.09 ± 0.37	3.92 ± 0.39	0.83	11.54(109.80)	[0.69, 0.97]	2.17
Common self-regulated writing	3.90 ± 0.38	3.41 ± 0.43	−0.49	−6.39(108.36)	[−0.64, −0.34]	−1.20
Prompts per task	7.58 ± 2.08	13.92 ± 3.37	6.35	11.99(91.57)	[5.29, 7.40]	2.25
AI text incorporation (%)	19.4 ± 7.1	42.7 ± 10.2	23.3	14.03(98.16)	[20.0, 26.6]	2.63
Independent revision ratio	0.74 ± 0.09	0.50 ± 0.11	−0.24	−12.64(105.85)	[−0.28, −0.20]	−2.37
Self-generated text proportion	0.78 ± 0.08	0.56 ± 0.11	−0.22	−12.10(100.46)	[−0.26, −0.18]	−2.27
Active drafting time (minutes)	48.2 ± 9.4	39.6 ± 10.7	−8.6	−4.52(108.20)	[−12.37, −4.83]	−0.85

### 4.4 Layer-specific offloading

Open collaboration exceeded bounded support at all four layers, with the largest differences at the idea and reasoning layers (Table 7).

**Table 7:** Layer-specific offloading within the AI-exposed subsample.

Layer	Bounded AI	Open AI	Difference	Welch *t*(*df*)	95% CI	Hedges' *g*
Surface	3.68 ± 0.46	4.10 ± 0.41	0.42	5.05(108.82)	[0.25, 0.58]	0.95
Structural	3.05 ± 0.49	3.92 ± 0.45	0.87	9.79(109.21)	[0.69, 1.05]	1.84
Idea	2.91 ± 0.44	3.88 ± 0.46	0.97	11.40(109.78)	[0.80, 1.14]	2.14
Reasoning	2.73 ± 0.47	3.79 ± 0.48	1.06	11.81(109.95)	[0.88, 1.24]	2.22

### 4.5 Prompt functions, adoption depth, and revision ownership

**Figure 1:**
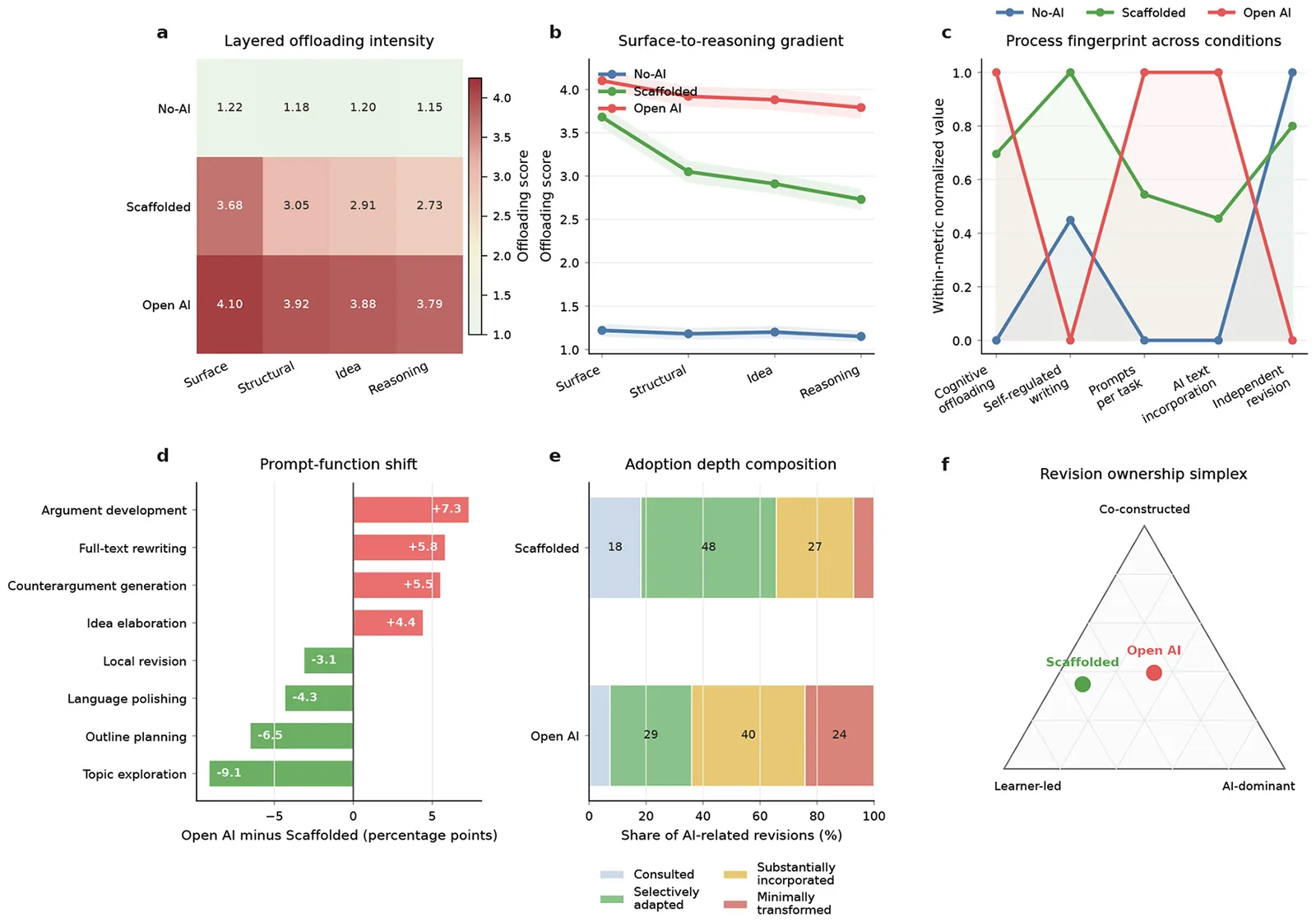
Layer-specific offloading and AI-use process profiles. Inferential comparisons of AI-use variables are restricted to the two AI-exposed conditions because no-AI values are structural zeros rather than naturally observed scores. **(a)** Layered offloading intensity. **(b)** Surface-to-reasoning gradient. **(c)** Process fingerprint across conditions. **(d)** Prompt-function shift. **(e**) Adoption depth composition. **(f)** Revision ownership simplex.

Prompt-function, adoption-depth, and revision-ownership profiles are summarized in Table 8 and visualized in Figure 1.

**Table 8:** AI-assisted process profiles across AI-supported conditions (%).

Dimension	Category	Bounded AI	Open AI
Prompt function	Topic exploration	19.8	10.7
	Outline planning	18.6	12.1
	Idea elaboration	15.4	19.8
	Argument development	11.2	18.5
	Counterargument generation	6.4	11.9
	Language polishing	14.7	10.4
	Local revision	10.9	7.8
	Full-text rewriting	3.0	8.8
Adoption depth	Consulted but not used	18.2	7.4
	Selectively adapted	47.5	28.6
	Substantially incorporated	27.1	39.8
	Minimally transformed	7.2	24.2
Revision ownership	Learner-led	54.6	26.8
	Co-constructed	34.8	39.5
	AI-dominant	10.6	33.7

### 4.6 Reflection and practice-volume checks

Within the bounded condition, stronger coded reflective engagement predicted higher Week 8 higher-order thinking after adjustment for baseline writing, HOT pretest, prior AI experience, and writing self-efficacy, *B* = 0.24, *SE* = 0.08, 95% CI [0.07, 0.41], *p* = 0.007, model *R*^2^ = 0.43. This within-condition association is consistent with a metacognitive mechanism but does not isolate the causal effect of compulsory reflection.

In the AI-exposed subsample, a joint model entered aggregate offloading and self-generated text proportion. Offloading remained negatively associated with Week 8 higher-order thinking, *B* = −0.37, *SE* = 0.08, *p* < 0.001, while self-generated text proportion was positively associated with the outcome, *B* = 0.74, *SE* = 0.28, *p* =.010. The adjusted open–bounded coefficient was no longer distinguishable from zero, *B* = 0.09, *SE* = 0.08, *p* = 0.255, and the model explained 66% of outcome variance. These results support reduced practice as a complementary rival mechanism rather than establishing a single pathway.

### 4.7 Exploratory associative decomposition within AI-exposed students

Open collaboration predicted higher aggregate offloading after covariate adjustment, *a* = 0.77, *SE* = 0.06, *p* < 0.001, mediator-model *R*^2^ = 0.67. Higher offloading was associated with lower Week 8 higher-order thinking, *b* = −0.45, *SE* = 0.08, *p* < 0.001. The adjusted total open–bounded contrast was *c* = −0.34, *SE* = 0.06, *p* < 0.001; after entering offloading, *c*′ = 0.00, *SE* = 0.08, *p* = 0.995. The indirect estimate was *ab* = −0.34, 95% bootstrap CI [−0.48, −0.21], and the outcome model explained 64% of the variance. The adjusted *a* path differs from the raw 0.83-point group difference because the model includes baseline writing, HOT pretest, prior AI experience, and writing self-efficacy.

Each row is a separate model with the same adjusted total contrast, *c* = −0.34 (*SE* = 0.06, *p* < 0.001). The results are associative decompositions embedded in a quasi-experimental contrast, not proof of causal mediation. The corresponding layer-specific associative decompositions are reported in Table 9.

**Table 9:** Full layer-specific associative decomposition within AI-exposed students.

Mediator	*a* (SE)	*p*_*a*_	$R_{M}^{2}$	*b* (SE)	*p*_*b*_	*c*^′^(SE)	$p_{c^{\prime }}$	$R_{Y}^{2}$	*ab*	95% CI
Surface	0.35 (0.08)	< 0.001	0.35	−0.24 (0.07)	0.001	−0.26 (0.06)	< 0.001	0.57	−0.08	[−0.14, −0.03]
Structural	0.81 (0.08)	< 0.001	0.57	−0.30 (0.06)	< 0.001	−0.10 (0.07)	0.160	0.61	−0.24	[−0.36, −0.12]
Idea	0.92 (0.08)	< 0.001	0.62	−0.21 (0.07)	0.001	−0.15 (0.08)	0.073	0.57	−0.20	[−0.33, −0.07]
Reasoning	1.00 (0.08)	< 0.001	0.65	−0.34 (0.06)	< 0.001	0.00 (0.08)	0.953	0.64	−0.34	[−0.47, −0.20]

### 4.8 Moderation by self-regulated writing

**Figure 2:**
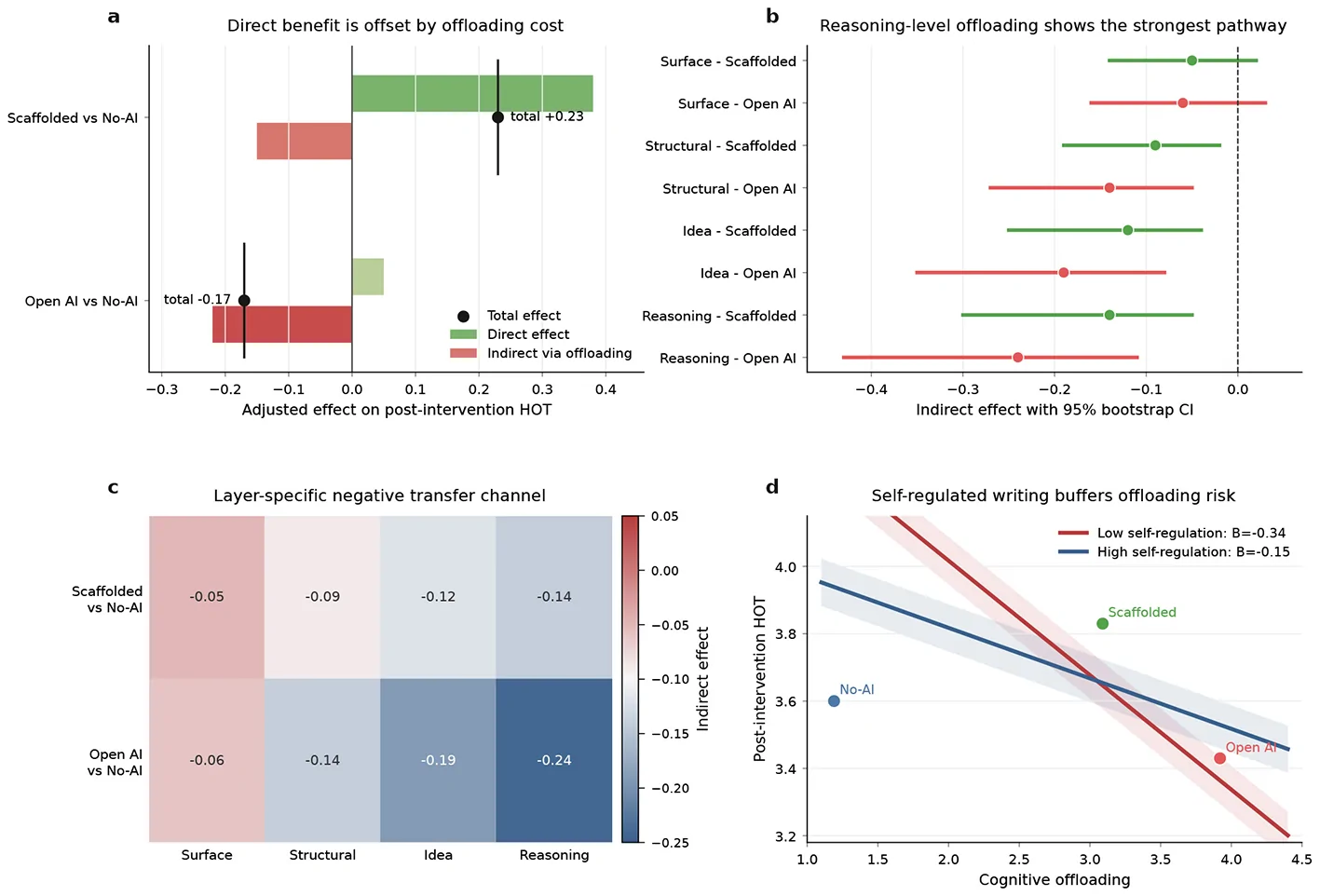
Exploratory mechanism analysis within the AI-exposed subsample. The paths represent adjusted associations. Idea and reasoning offloading show the largest negative indirect estimates, while higher self-regulated writing attenuates the negative offloading association. **(a)** Direct benefit is offset by offloading cost. **(b)** Reasoning-level offloading shows the strongest pathway. **(c)** Layer-specific negative transfer channel. **(d)** Self-regulated writing buffers offloading risk.

Among AI-exposed students, common self-regulated writing had *M* = 3.66 and *SD* = 0.47. The offloading-by-SRW interaction was positive, *B* = 0.22, *SE* = 0.11, *p* = 0.043, model *R*^2^ = 0.66. The conditional offloading slope was −0.44 at mean SRW, 95% CI [−0.58, −0.29]; −0.54 at one SD below the mean, 95% CI [−0.72, −0.36]; and −0.33 at one SD above the mean, 95% CI [−0.51, −0.15]. The arithmetic is consistent with the interaction: moving from one SD below to one SD above the mean changes the slope by approximately 2(0.47)(0.22) = 0.21. These exploratory mechanism results are summarized in Figure 2.

### 4.9 Week 8 independent no-AI outcomes

The bounded condition had the highest observed Week 8 means (Table 10). Student-level bounded–open effect sizes were *g* = 0.83 for writing quality, 1.07 for higher-order thinking, 1.21 for argument depth, and 1.17 for independent revision quality. These are descriptive effect sizes; cluster-sensitive estimates provide the principal uncertainty assessment. The corresponding class-clustered bounded-open contrasts are reported in Table 11.

**Table 10:** Week 8 independent no-AI near-transfer outcomes.

Condition	Writing quality	Higher-order thinking	Argument depth	Independent revision quality
No-AI writing	3.66 ± 0.35	3.60 ± 0.37	3.57 ± 0.39	3.62 ± 0.36
Bounded AI support	3.88 ± 0.31	3.83 ± 0.34	3.86 ± 0.33	3.88 ± 0.32
Open AI collaboration	3.59 ± 0.38	3.43 ± 0.39	3.40 ± 0.41	3.46 ± 0.39

**Table 11:** Class-clustered bounded–open contrasts for Week 8 outcomes.

Outcome	Adjusted difference	Clustered SE	*t*(5) 95% CI	Wild-cluster *p*	Interpretation
Writing quality	0.27	0.02	[0.21, 0.32]	0.050	Direction stable; boundary-level uncertainty
Higher-order thinking	0.35	0.06	[0.19, 0.51]	0.063	Direction stable; imprecise
Argument depth	0.42	0.06	[0.27, 0.58]	0.054	Direction stable; imprecise
Revision quality	0.39	0.05	[0.27, 0.51]	0.053	Direction stable; imprecise

**Figure 3:**
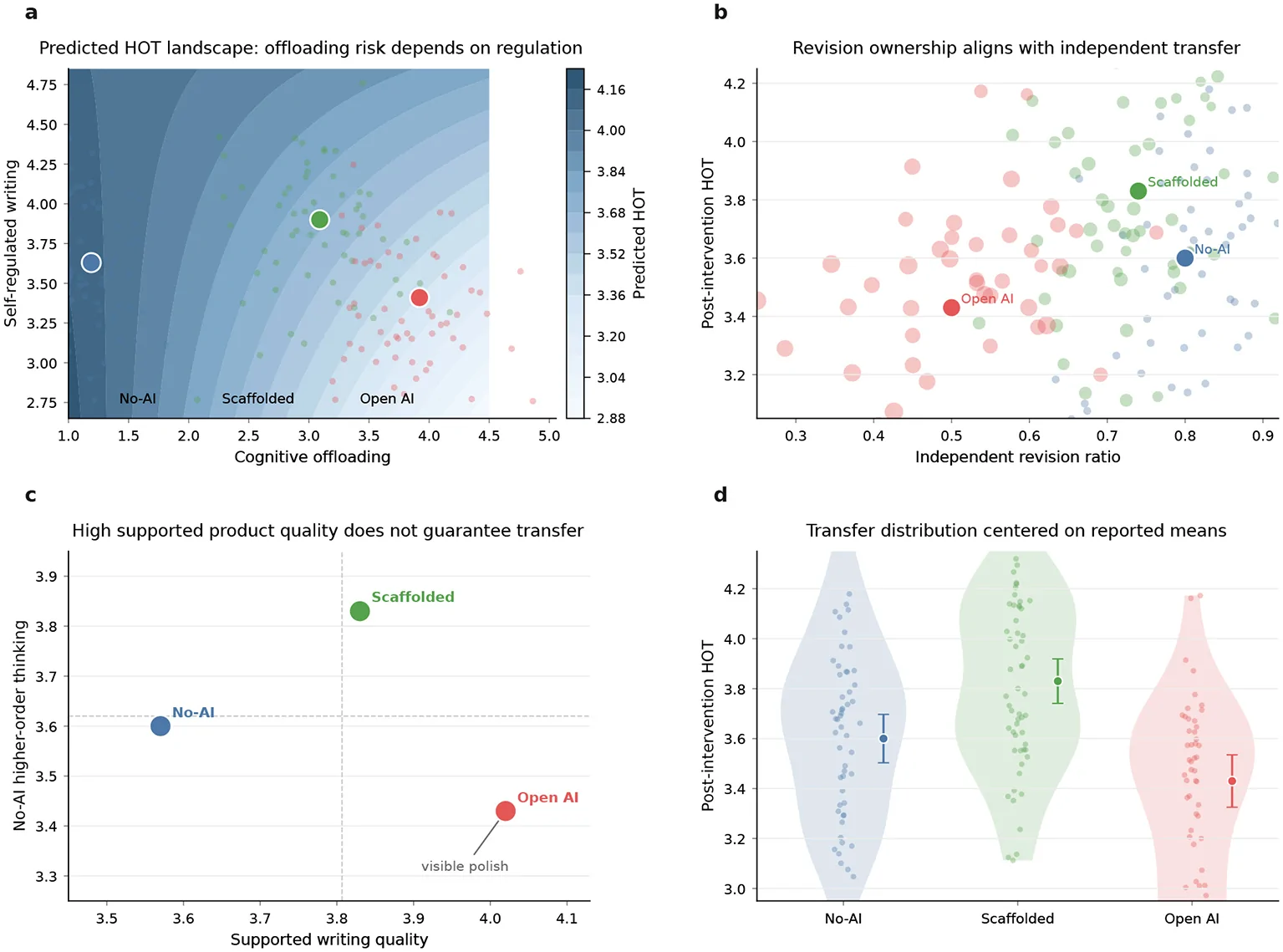
Supported-performance and independent-performance landscape. The figure contrasts intervention writing quality with Week 8 independent higher-order thinking and relates the observed outcome pattern to offloading, self-regulated writing, self-generated text, and revision ownership. **(a)** Predicted HOT landscape: offloading risk depends on regulation. **(b)** Revision ownership aligns with independent transfer. **(c)** High supported product quality does not guarantee transfer. **(d)** Transfer distribution centered on reported means.

The consistent direction and magnitude of the bounded–open contrasts support the study's central pattern, but the wild-cluster results show that exact significance claims are fragile with only six classes. The evidence is therefore framed around estimated differences, intervals, process convergence, and design limitations rather than a binary set of student-level *p* values. The contrast between supported writing quality and independent Week 8 performance is summarized in Figure 3.

### 4.10 Demand characteristics and textual examples

Two intervention prompts concerned AI and online learning. These topics could cue students to discuss the study's underlying concern and could permit raters to infer condition despite de-identification. Complete prompts are disclosed in the Supplementary Material. Textual examples are used only to illustrate coding categories and are not treated as independent confirmation of the hypotheses.

## 5 Discussion

### 5.1 Supported performance and independent performance diverged

The central finding is a divergence, not a general verdict that GenAI is beneficial or harmful. Open collaboration showed the highest observed writing quality while AI support was available, particularly in aspects that a language model can directly improve. Yet bounded support showed the highest observed performance after AI was removed. This pattern aligns with the distinction between effects achieved *with* technology and effects that remain *of* technology, as well as the distinction between performance during practice and learning demonstrated later (Salomon et al., 1991; Soderstrom and Bjork, 2015).

The class-sensitive analysis materially qualifies this conclusion. The open–bounded supported-performance contrast had a wide six-cluster uncertainty profile, and all four Week 8 exact wild-cluster *p* values lay at the 0.050–0.063 boundary. The defensible statement is therefore that a consistent, practically substantial classroom pattern favored open collaboration during support and bounded support during independent assessment. The design does not establish precise individual-level causal effects.

### 5.2 Delegation depth, reflection, and practice were jointly relevant

The process data explain why a binary AI/no-AI contrast is inadequate. Open collaboration was associated with much larger increases in idea and reasoning offloading than in surface support, greater incorporation of AI text, and more AI-dominant revision. The AI-only associative decompositions linked deeper offloading to weaker independent higher-order thinking, with the largest indirect estimates for idea and reasoning layers.

Two rival explanations remain active. First, bounded support included compulsory reflection. Reflective engagement predicted Week 8 performance within that condition, but the analysis cannot separate reflection from delegation boundaries. Second, open-AI students generated less of their own text and spent less active time drafting. In the joint model, both offloading and self-generated text contributed to Week 8 performance. The most defensible mechanism account is therefore complementary: unrestricted delegation may reduce both the cognitive processing of ideas and the amount of deliberate composing practice.

### 5.3 Self-regulated writing reduced but did not remove the association

Higher self-regulated writing weakened the negative association between offloading and independent higher-order thinking. Students who planned, monitored coherence, evaluated suggestions, and retained revision authority appeared better able to use AI without fully surrendering cognitive responsibility. The offloading slope remained negative even at one SD above mean SRW, however. Metacognitive skill should not be treated as a complete substitute for opportunities to perform the reasoning operations independently.

### 5.4 Instructional implications

The results support concrete design choices. Students can complete an independent claim–evidence plan before consulting AI. AI access can initially be limited to diagnostic questions, alternative options, reader feedback, and local language clarification. Students can submit pre-AI and post-AI versions and classify each AI-influenced change as accepted, modified, or rejected with a rationale. Rubrics can allocate credit to reasoning provenance, evidence evaluation, and revision ownership rather than final fluency alone. Support can then be faded: early tasks may allow structured prompting, later tasks may restrict AI to feedback, and periodic independent tasks can assess what remains after assistance is removed.

These practices operationalize self-regulated learning through goal setting, monitoring, evaluation, and strategic revision. They also align more closely with contingency and transfer-of-responsibility principles than unrestricted collaboration does.

### 5.5 Limitations

The principal limitation is the six-class allocation. Condition remained partially confounded with class, timetable, and cohort, and wild-cluster inference was fragile. Second, bounded support combined delegation limits with compulsory reflection. Third, Week 8 measured immediate same-course, same-genre near transfer rather than delayed, cross-genre, or far transfer. Fourth, AI- and online-learning topics created possible demand characteristics and imperfect content-based blinding. Fifth, the offloading framework showed promising initial structural, reliability, and convergence evidence but requires independent validation. Sixth, self-generated text and active drafting time were derived from available records rather than captured through complete keystroke logging. Finally, the sample comprised Chinese L1 students writing in English at one university.

## 6 Conclusion

The strongest AI-supported products were not followed by the strongest independent no-AI performance. Open collaboration showed the highest observed writing quality while support was present, whereas bounded support combined with reflective accountability showed the most favorable Week 8 profile. The process evidence indicates that deeper delegation and reduced independent composing practice are plausible, overlapping explanations.

These findings should be interpreted as a coherent classroom pattern rather than a definitive causal estimate. Even so, they support a practical educational principle: GenAI can assist writing without assuming responsibility for the claims, evidence interpretations, warrants, counterarguments, and revision decisions through which students develop independent competence. Evaluation should therefore consider both the quality of the final text and the cognitive work that remains attributable to the learner.

## Data Availability

The raw data supporting the conclusions of this article will be made available by the authors, without undue reservation.
